# An exploration of anti-corruption and health in international organizations

**DOI:** 10.1371/journal.pone.0269203

**Published:** 2022-08-02

**Authors:** Andrea Bowra, Gul Saeed, Ariel Gorodensky, Jillian Clare Kohler

**Affiliations:** 1 Dalla Lana School of Public Health, University of Toronto, Toronto, Canada; 2 WHO Collaborating Centre for Governance, Accountability and Transparency in the Pharmaceutical Sector, University of Toronto, Toronto, Canada; 3 Leslie Dan Faculty of Pharmacy, University of Toronto, Toronto, Canada; 4 Munk School of Global Affairs and Public Policy, University of Toronto, Toronto, Canada; University of Glasgow, UNITED KINGDOM

## Abstract

Corruption is a global wicked problem that threatens the achievement of health, social and economic development goals, including Sustainable Development Goal # 3: Ensuring healthy lives and promoting well-being for all. The COVID-19 pandemic and its resulting strain on health systems has heightened risks of corruption both generally and specifically within health systems. Over the past years, international organizations, including those instrumental to the global COVID-19 response, have increased efforts to address corruption within their operations and related programs. However, as attention to anti-corruption efforts is relatively recent within international organizations, there is a lack of literature examining how these organizations address corruption and the impact of their anti-corruption efforts. This study addresses this gap by examining how accountability, transparency, and anti-corruption are taken up by international organizations within their own operations and the reported outcomes of such efforts. The following international organizations were selected as the focus of this document analysis: the World Health Organization, the Global Fund, the United Nations Development Programme, and the World Bank Group. Documents were identified through a targeted search of each organization’s website. Documents were then analyzed combining elements of content analysis and thematic analysis. The findings demonstrate that accountability and transparency mechanisms have been employed by each of the four international organizations to address corruption. Further, these organizations commonly employed oversight mechanisms, including risk assessments, investigations, and audits to monitor their internal and external operations for fraud and corruption. All organizations used sanction strategies meant to reprimand identified transgressors and deter future corruption. Findings also demonstrate a marked increase in anti-corruption efforts by these international organizations in recent years. Though this is promising, there remains a distinct absence of evidence demonstrating the impact of such efforts on the prevalence and severity of corruption in international organizations.

## Introduction

Corruption is a “global wicked problem” meaning it is a complex problem that involves a high number of stakeholders and is deeply intertwined with other social and cultural issues [1]. Corruption limits access to healthcare, hampers health equity [2], and threatens the achievement of human rights [3]. According to Transparency International (TI), corruption is defined as “the abuse of entrusted power for private gain” [4]. What is more, corruption leads to inefficiencies in the expenditure of public funds [5], stifles trust in public institutions [6], and undermines democracy [4]. Corruption most prominently affects poor and marginalized populations, further exacerbating inequities [7] and hindering social and economic development [2, 5, 8–10]. According to the United Nations (UN) Secretary General, the financial cost of corruption is estimated to be approximately 5% of the global Gross Domestic Product [11]; thus, “if all countries were to reduce corruption…they could gain $1 trillion in lost tax revenues” [6].

The health sector is especially vulnerable to corruption as it is technically complex, has a large number of stakeholders, and has high financial stakes [12]. According to TI, nearly $500 billion USD, which amounts to 7% of global healthcare expenditure at an amount sufficient to achieve Universal Health Coverage, is lost to corruption each year [7]. Furthermore, nearly 140,000 annual child deaths are associated with corruption [13]. In low- and middle- income countries (LMICs), more than 80% of individuals have experienced healthcare corruption [14].

Due to its associated high financial burden, impact on public service provision, and negative health outcomes, corruption has been acknowledged as a significant threat to achieving the Sustainable Development Goals (SDGs), specifically SDG 3, “Ensure healthy lives and promote well-being for all at all ages,” and SDG 16, “Promote peaceful and inclusive societies for sustainable development, provide access to justice for all and build effective, accountable and inclusive institutions at all levels,” which includes target 16.5: “Substantially reduce corruption and bribery in all their forms” [15, 16]. In light of the COVID-19 pandemic, corruption poses an even higher threat to the achievement of these and other SDGs. The COVID-19 pandemic has amplified the health sector’s susceptibility to corruption by increasing demand for the limited supply of vaccines, medicines, diagnostics, and health care services [17]. Numerous cases of corruption have been recorded, including falsified COVID-19 diagnostics [18], personal gain from vaccine deals [19], and price gouging of COVID-related health products [20].

Ultimately, corruption hinders the equitable distribution of COVID-related health products and hampers efforts to control virus spread. International Organizations (IOs) such as the World Health Organization (WHO) and the United Nations Development Programme (UNDP) have been committed to anti-corruption for nearly three decades. Recently, they have played key roles in the international COVID-19 response, which has highlighted the pernicious implications of corruption on global health systems and as a result, has strengthened IOs’ commitments to promoting and facilitating anti-corruption in the health sector.

The engagement of IOs in anti-corruption began in the mid-1990s with what Moisés Naim described as a “corruption eruption”: an increased awareness of corruption and efforts to address this issue [21]. In 1996, in a critical turning point, the World Bank President, James Wolfensohn, gave his famous “cancer of corruption” speech, facilitating the acknowldgement of corruption as an issue that international organizations need to acknowledge and address [22].

International relations and other disciplinary scholars have indeed examined the emergence of a global anti-corruption norm which first involved raising awareness of the issue of corruption in global systems, then the acceptance and institutionalization of anti-corruption commitments through legal and policy instruments, and finally, the acceptance of the anti-corruption norm by the vast majority of relevant actors. Despite these achievements towards anti-corruption goals, scholars such as Kaufmann [23] and Rose-Ackerman [24] question the norm’s long-term potential without increased accountability structures in place for anti-corruption practices. Further, Bukovansky [25] critiques the anti-corruption discourse as being hollow as corruption discourses are often linked to the problem of “under-development,” meaning that “more developed” countries, IOs, and non-governmental organizations (NGOs) often claim the moral high ground for themselves.

In relation to the uptake of anti-corruption by IOs specifically, existing scholarship in the field demonstrates the role that IOs play in fighting corruption [26–28] and the need for transparency, accountability, and good governance within them [26, 29]. Of significance, Berkman et al. [27] explored the progress IOs have made in the fight against corruption, concluding that though there has been a rapidly expanded interest in and uptake of anti-corruption efforts, IOs must critically examine whether their commitments to anti-corruption are authentic and effective. However, fourteen years later, we have yet to determine the authenticity and efficacy of IOs’ commitments to anti-corruption. While Berkman et al.’s research offers a strong base from which to examine IOs’ commitments, approaches, and actions against corruption, there is a significant absence of literature on how IOs address corruption specifically within the health sector. As described above, this is critical as we continue to grapple with the increased strain that the ongoing COVID-19 pandemic has put on health systems globally.

## Objectives of paper

To address this gap, this document analysis provides an initial exploration of how anti-corruption, transparency and accountability (ACTA) are taken up by IOs within their own operations, and the reported outcomes of these efforts. We describe and compare four IOs’ approaches to anti-corruption both generally and within the health sector specifically: the WHO, UNDP, the Global Fund to Fight AIDS, Tuberculosis and Malaria (Global Fund), and the World Bank Group (World Bank). This will be achieved through the exploration of the following interrelated research questions:

1. How do IOs working in the health sector position themselves in relation to the issue of corruption?
2. Given that transparency and accountability are acknowledged as best practices for addressing corruption, how are these approached within IOs’ anti-corruption mechanisms and institutions?
3. What oversight mechanisms are employed as a means of addressing corruption?
4. What systems exist to enforce anti-corruption efforts and address identified cases of corruption?
5. How are IOs monitoring and evaluating anti-corruption efforts and what are their reported outcomes?

## Materials and methods

### Study design

This study builds on a targeted website review conducted between September and October of 2019 which aimed to provide a brief overview of how IOs address corruption within their operations [26]. For this research, the WHO, the World Bank, the Global Fund, and UNDP were selected as focus. This selection was guided by criterion-based sampling (also referred to as purposive sampling) [30]. In criterion-based sampling, units are selected because of their features that enable exploration and understanding of the central themes the researcher wishes to examine [30]. Criteria for selection are informed by factors including the principle aim of the study, existing knowledge of the field, hypotheses, and/or gaps in knowledge. In coherence with this logic, the four IOs above were selected as focus of this study as they not only play an integral role in global health systems (see Table 1 for the organizations’ health-related roles or mandates), including global responses to the COVID-19 pandemic, but they have recently strengthened their commitments to anti-corruption through their participation in and formation of the Coalition for Accountability, Transparency, and Anti-Corruption in Health (CATCH). CATCH aims to support countries in mitigating risks of corruption in the health sector [31, 32].

**Table 1:** IOs’ health-related mandates or roles.

Organization	Health-Related Role or Mandate
UNDP	“In line with its Strategic Plan 2018–2021 and HIV, Health & Development Strategy 2016–2021, UNDP’s work on health contributes to its broader commitment to eradicate poverty, reduce inequalities, strengthen effective and inclusive governance, and build resilient and sustainable systems for health” [33]
Global Fund	“The Global Fund is a partnership designed to accelerate the end of AIDS, tuberculosis and malaria as epidemics” [34]
WHO	“Dedicated to the well-being of all people and guided by science, the World Health Organization leads and champions global efforts to give everyone, everywhere an equal chance to live a healthy life” [35]
World Bank	“The World Bank Group works to help nations build healthier, more equitable societies and to improve fiscal performance and country competitiveness” [36]

Importantly, this case selection is not meant to be representative of the universe of IOs. Rather, UNDP, Global Fund, WHO and World Bank are all members of CATCH, as well as the only existing IOs who have a history of addressing corruption in the global health sector, and therefore provide a strong base from which to explore the aforementioned research questions.

**Fig 1:**
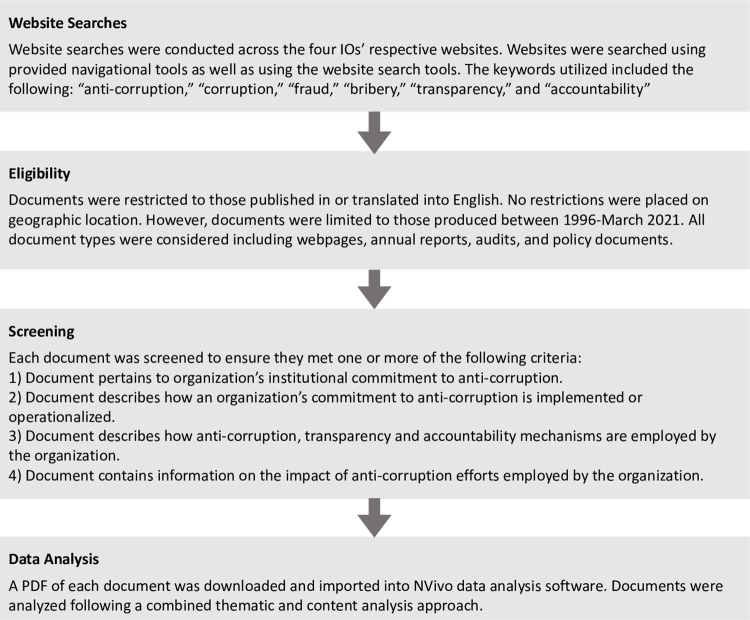
Document analysis methods.

To achieve our aim of examining anti-corruption efforts in the WHO, World Bank, Global Fund, and UNDP, we conducted a document analysis of publicly available materials produced by the selected IOs. Document analyses are a valuable tool in qualitative research as they facilitate intensive studies that produce rich descriptions of phenomena, events, programs, or in our case, organizations [37, 38]. Public records are a crucial source of information as they provide ongoing records of an organization’s activities and can include mission statements, annual reports, policy manuals, strategic plans, and program evaluations [39]. See Fig 1 for a visualization of study methods.

### Data collection

Website searches were conducted initially between September and October of 2019 and then again in March of 2021. Websites were searched following their provided navigation menus as well as through searches using the following key words: corruption, anti-corruption, accountability, transparency, governance, oversight, audit, and evaluation. No restrictions were placed on geographic location, though searches were limited to materials published between 1996 (the year World Bank President, James Wolfensohn, gave his famous “Cancer of Corruption” speech [22] and March 2021, to ensure the most relevant and current publications were captured. Documents were screened as they were identified to ensure they met the following eligibility criteria: (1) documents either pertained to organizations’ institutional commitment to anti-corruption OR described how this commitment is implemented/operationalized OR described how ACTA mechanisms are employed OR contained information on the impact of anti-corruption efforts; and (2) documents were in English or could easily be translated into English. The documents extracted included organizational reports, policies, and internal and external audits. The variety in type of documents extracted was intentional because it is recommended that a wide array of documents are accessed when using the document analysis methodology in order to collect a preponderance of evidence [40]. It is important to note that, while fraudulent acts are not necessarily acts of corruption, and vice versa, fraud often accompanies and leads to corruption [41]. The studied IOs, however, use the terms fraud and corruption interchangeably; this is reflected in the findings presented below.

### Data analysis

A PDF of each document retrieved through the searches was downloaded and imported into NVivo 12 data analysis software. Documents were then analyzed by three research team members combining elements of content analysis and thematic analysis [40]. According to Bowen (2009), content analysis, commonly employed in document analyses, is the process of organizing information into categories related to the central questions of the research [40]. Accordingly, data was categorized based on Vian’s [42] concepts, frameworks, and approaches for ACTA in global health. This comprehensive review produced the following categories that guided our initial categorization of data: anti-corruption approaches, transparency interventions, risk assessments, community monitoring, whistleblowing and complaint mechanisms, and audits. Categories were amended in an iterative fashion throughout data analysis to better reflect the mechanisms, strategies, and interventions identified. The final list of categories into which the data was organized and presented below included: anti-corruption approaches; transparency and accountability interventions; organizational oversight, which included risk assessments and audits and investigations; and enforcement and sanctions. As our research also aimed to examine the reported outcomes of anti-corruption efforts, this category was added for analysis as well. Once data was categorized, data within categories was coded following a thematic analysis which is a means of recognizing patterns and emerging themes pertinent to each category [40, 43]. Codes were amended in an iterative fashion throughout the analysis process to ensure they were reflective and comprehensive of the terminology employed across the range of documents.

## Results

For this study, 100 documents were included in the final analysis: 36 documents from UNDP, 21 from the World Bank, 28 from the Global Fund, and 15 from the WHO. In total, analysis was conducted on 11 audits, 3 annual reports, 3 terms of reference, 8 documents about institutions rules/regulations/codes of conduct, 17 organizational reports, 17 institutional policies, and 41 miscellaneous documents. Documents varied significantly in page-length, ranging from 2–187 pages. 43 of the total documents pertained to anti-corruption in the health sector specifically.

Overall, 34% of the documents had information about oversight (internal and external), 11% had information related to anti-corruption policy or miscellaneous information about anti-corruption, 7% had information about capacity building, 4% had information about the impact of anti-corruption measures, 9% had information about accountability, 8% had information about transparency, and 3% had information about the use of technology in anti-corruption. The remaining 24% had information about corruption prevention, collaboration for anti-corruption, corruption reporting mechanisms, examples of corruption, information sharing, the enforcement of anti-corruption measures, sanctions, anti-corruption during COVID-19, good governance, and IO position statements on anti-corruption. In general, documents related to more than one topic. For example, the Global Fund’s Office of the Inspector General 2018 Annual Report had information about oversight, the impact of the Fund’s anti-corruption work, and capacity building, and thus was coded for each of these topics [44].

### Anti-corruption approaches

To understand how corruption is being addressed by IOs within their own operations and related programs, the research team summarized and compared the anti-corruption approaches, institutions, and mechanisms found within IO documents. The findings are presented below, beginning with anti-corruption position statements, then transparency and accountability interventions, followed by oversight mechanisms including risk assessments and investigations and audits, enforcement and sanctions, and ending with a discussion of the reported impacts of these anti-corruption efforts.

#### Position statements

A position statement on corruption informs the public about an organization’s stance on corruption and highlights the values or principles guiding the organization’s operations. As seen in Table 2, the WHO implements a zero-tolerance approach to fraud across the organization. Similarly, the Global Fund, the world’s largest public health financer, has zero tolerance for fraud and corruption as these result in the wastage of essential resources, preventing resources from reaching people who need them [45–47]. Likewise, UNDP has implemented a zero-tolerance policy for fraud and corruption that applies to UNDP staff members and non-staff personnel, vendors, and implementing partners [48]. *UNDP Policy against Fraud and other Corrupt Practices* defines corruption as “the act of doing something with an intent to give an advantage inappropriate with official duties to obtain a benefit, to harm or to influence improperly the actions of another party” [48]. UNDP posits that anti-corruption is key to advancing sustainable development outcomes [49]. The World Bank has also advanced a zero-tolerance approach to corruption, recognizing that corruption poses a serious challenge to achieving its objective of eradicating extreme poverty by 2030 [50]. The World Bank defines a corrupt practice as “the offering, giving, receiving, or soliciting, directly or indirectly, of anything of value to influence improperly the actions of another party” [51]. However, it acknowledges that corruption can take different forms and have various impacts.

**Table 2:** Defining and approaching anti-corruption.

	UNDP	Global Fund	WHO	World Bank
**Position Statement**	Zero tolerance for fraud and corruption.	Zero tolerance for fraud and corruption.	Zero tolerance for fraud.	Zero tolerance for corruption.
**Definition of Corruption**	“The misuse of public power, office or authority for private benefit–through bribery, extortion, influence peddling, nepotism, fraud, speed money or embezzlement. Although corruption is often considered a sin of government and public servants, it also prevails in the private sector” [52]	“A Corrupt Practice is the offering, giving, receiving or soliciting, directly or indirectly, of anything of value to influence improperly the actions of another party” [53].	Employs TI’s definition of corruption as “the abuse of entrusted power for private gain” [31]	“A corrupt practice is the offering, giving, receiving, or soliciting, directly or indirectly, of anything of value to influence improperly the actions of another party” [51]

#### Transparency & accountability interventions

Given that transparency and accountability are acknowledged as best practices for addressing corruption, these were included as standard principles across all IOs in their anti-corruption policies and mechanisms. TI describes transparency as “knowing the who, why, what, how and how much. [Transparency] means shedding light on formal and informal rules, plans, processes and actions” [4]. Transparency is a crucial anti-corruption tool as it can help the public hold governments, companies, and organizations accountable for their actions. It is, however, important to note that simply making information available does not suffice. Rather, information needs to be accessible, understandable, and usable by the public. In terms of accountability, Vian and Kohler [54] explain that accountability mechanisms are essential to anti-corruption work as they are what make institutions responsive to their respective publics. Accountability is vital to transparency mechanisms as it allows for possible consequences when corrupt activities are identified. Thus, anti-corruption interventions often employ transparency and accountability mechanisms in tandem.

#### Transparency

Initiatives to improve transparency employed by IOs took two main forms (see Table 3 for summary). The first was information sharing, where operational information, including funding, audits, and investigation reports, were published online in a publicly available format. The second form, which overlaps with accountability, was capacity building initiatives targeting civil society to encourage engagement in transparency and accountability initiatives. Across organizations, transparency mechanisms were limited by a lack of inter- and intra- agency coordination, specifically in countries that were post-conflict or in political transition. In these countries, it was also more difficult to facilitate country ownership of transparency initiatives.

**Table 3:** Defining and operationalizing transparency.

	UNDP	Global Fund	WHO	World Bank
**Definition**	“A process by which reliable, timely information about existing conditions, decisions and actions relating to the activities of the organization is made accessible, visible and understandable” [55].	None found.	None found.	None found.
**Representative Mechanisms**	• Evaluation Resource Centre • Program for Accountability and Transparency (PACT) • Information Disclosure Policy	• Documents Policy and Policy for Disclosure of Reports • Wambo.org	• Measuring Transparency in the Public Pharmaceutical Sector	• World Bank’s Fiscal Openness Working Group • Public Participation Principles • Guidance on Fiscal Transparency

As seen in Table 3, UNDP was the only IO in this study to provide a definition of transparency, defining it as “a process by which reliable, timely information about existing conditions, decisions and actions relating to the activities of the organization is made accessible, visible and understandable” [55]. UNDP initially began addressing the need for transparency within their operations and programs in 1997 through their *Programme for Accountability and Transparency* (PACT). This was taken up with renewed focus in the *Global Anticorruption Initiative* (GAIN) in 2014 [49]. Transparency initiatives focused mainly on the use of information and communications technologies, as well as access to information movements including those implemented through the *United Nations Convention against Corruption* (UNCAC). Within their own operations, UNDP has multiple transparency mechanisms, and consistently ranks among the top organizations in the Aid Transparency Index [56], an independent measure of aid transparency of major development agencies [56]. The Aid Transparency Index provides donors, governments, and the public with information about initiatives and their outcomes to increase efficiency and accountability for achieving health and development outcomes. Internal transparency mechanisms within UNDP include their *Information Disclosure Policy*, which commits the organization to making information about their programs and operations publicly available [57], and UNDP Transparency Portal, where such information is publicly accessible.

The Global Fund, like UNDP, consistently ranks among the top organizations on the Aid Transparency Index [56]. Their internal *Documents Policy* and *Policy for Disclosure of Reports*, issued by their Office of the Inspector General (OIG), requires the publishing of internal and external assurances, investigations, and OIG Board reports. The Global Fund publishes this information on public websites as well as on social media through their twitter account. However, their policy also recognizes that there may be circumstances where legal or practical constraints require restricted disclosure. To support transparency in its funded programs, the Global Fund launched wambo.org, an online purchasing platform that supports transparency in procurement processes by providing implementers with instant access to price comparisons, lead times, and supply quantities across suppliers [58]. The Global Fund states that this website increases transparency by giving both the suppliers and bidders greater process visibility, as well as aims to improve the availability of products, provide better prices, and reduce procurement costs.

The WHO highlights transparency in both research and procurement processes through their code of ethics, which states that the organization is committed to “high quality research that is ethical, expertly reviewed, efficient, accessible, transparent, carefully monitored and rigorously evaluated” [59]. Their procurement processes are similarly designed to promote transparent competition, though the specific strategies in place to do so were not explicitly described in the documents. Finally, in terms of promoting transparency among their member states, the WHO created an assessment instrument entitled *Measuring Transparency in the Public Pharmaceutical Sector* as part of their *Good Governance for Medicines Program* [60]. This tool provides a practical methodology to measure the level of transparency and vulnerability to corruption in key processes of a country’s pharmaceutical system, as well as the means to monitor progress of initiatives over time.

The World Bank committed to increasing transparency as part of its efforts to combat corruption at the *Anti-Corruption Summit in 2016*. The World Bank does this by providing technical assistance via its *Public Participation Principles* and *Guidance on Fiscal Transparency* documents. It further supports countries in implementing transparency pacts and other forms of collective action to support transparency around transactions. As the World Bank relies heavily on their audit, investigation, and sanction systems to address corruption, it further states that these systems are instrumental in ensuring transparency and accountability in financial management [61]. The World Bank has played a role in creating international transparency standards, open contracting standards, and asset disclosure standards, and promotes these acts as steps towards providing leadership in transparency.

Interestingly, all IOs in this study promoted the use of technological innovations as a means of promoting transparency within their organizations and among member/recipient states. Such innovations can provide wide access to essential information; however, it is important to note that citizen engagement is necessary for transparency mechanisms to be effective in holding organizations and governments accountable for their actions.

#### Accountability

The Global Fund, UNDP, and the WHO explicitly define accountability as seen in Table 4 and have created and implemented accountability frameworks within their operations. The three definitions are broadly in agreement that accountability is the responsibility or answerability for one’s actions and decisions. Accountability efforts across all institutions faced similar barriers and facilitators. Barriers that undermined accountability initiatives included political impetus, government commitment and inclination to political reforms, and the limited scope of IOs’ interventions. Furthermore, accountability efforts are often hindered by existing governance structures and a lack of available resources. Accountability measures overall saw the most success when they took a sectoral approach as opposed to a more generalized approach.

**Table 4:** Defining and operationalizing accountability.

	UNDP	Global Fund	WHO	World Bank
**Definition**	“The obligation to (i) demonstrate that work has been conducted in accordance with agreed rules and standards and (ii) report fairly and accurately on performance results vis-à-vis mandated roles and/or plans” [55].	“Accountability entails responsibility for one’s activities and decisions. It also includes the duty to immediately inform the Global Fund of possible ethical misconduct in Global Fund Operations” [62].	“The obligation of every member of the Organization to be answerable for his/her actions and decisions, and to accept responsibility for them. WHO is accountable to its Member States, and WHO staff are accountable for achieving objectives and results in accordance with the Programme Budget and with regulations, rules and standards” [59].	None found.
**Representative Mechanisms**	• UNDP Accountability Framework • Technical support to countries to develop their own accountability frameworks	• Performance and Accountability Framework	• Accountability Framework	• Audits and Investigations • Sanctions System

UNDP began addressing the need for accountability in its operations and partner countries in the early 1990s through accountability, transparency, and integrity programs. These programs were solidified in UNDP’s corporate policy paper *Fighting Corruption to Improve Governance*, published in 1998, and again in their 2004 *Anti-Corruption Practice Note*. UNDP’s current accountability system is composed of an accountability framework and an oversight policy, both of which “underscore the commitment of UNDP to results and risk-based performance management, as well as the shared values of accountability and transparency” [63]. In addition to their portfolio of internal accountability mechanisms, UNDP provides technical assistance within its governance portfolio by supporting countries to develop their own accountability frameworks. One example is a training workshop held in partnership with UNDP’s Regional Project on Anti-Corruption and Integrity in the Arab Countries (UNDP-ACIAC), the Jordanian Anti-Corruption Commission, and the Arab Anti-Corruption and Integrity Network (ACINET).

In the documents reviewed, the Global Fund’s accountability mechanisms were not detailed as robustly as UNDP’s, however, it does have a *Performance and Accountability Framework* in place to govern internal operations. The *Performance and Accountability Framework* emphasizes “improving organizational efficiency, streamlining business processes and systems, and promoting agile decision-making and a stronger focus on delivery and performance management” [64]. As demonstrated by this aim, the Global Fund mobilizes the concept of accountability largely to refer to internal reporting structures with a focus on ensuring efficient work processes. At the country level, the Global Fund states that it aims to create personal accountability with its partners by referring findings from their internal audits and investigations to national law enforcement agencies.

Like other IOs, the WHO has issued an *Accountability Framework* [65]. Similar to the Global Fund, the WHO addresses accountability as it relates to the clear delineation of roles and responsibilities across the organization to increase efficiency.

Lastly, the World Bank operationalizes accountability through audits and sanctions. Former World Bank President, Robert B. Zoellick, stated that by “holding companies and individuals accountable through a fair and robust process, the Bank Group’s sanctions system promotes integrity and levels the playing field for those committed to clean business practices” [66]. Furthermore, at the *2016 Anti-Corruption Summit*, the World Bank committed to providing support at the country level to help clients develop accountable and transparent institutions. In the context of the COVID-19 pandemic, the World Bank’s *Governance and Accountability Actions Plans* build on existing fiduciary agreements while engaging civil society organizations to help monitor COVID-19 response projects as a measure to strengthen accountability.

### Organizational oversight

Oversight mechanisms are defined broadly as mechanisms in place, either within or external to an organization, to detect corruption within organizational operations and associated programming. Oversight mechanisms across IOs generally followed similar structures, including risk assessment frameworks, investigations, and audits as evidenced in Table 5. These oversight mechanisms are responsible for identifying potential and actual corruption within the organizations’ operations, programs, and funded partners. While all IOs have internal audit and investigations units, they also periodically engage external auditors to assess the efficiency and efficacy of their internal processes.

**Table 5:** Oversight mechanisms.

	UNDP	Global Fund	WHO	World Bank
**Risks Assessments**	Enterprise Risk Management System (ERM) is responsible for identifying risk across all levels of the organization. Project Managers assume responsibility for identifying risk during project development phases.	Office of Inspector General (OIG) conducts risk assessments referred by the Secretariat. They provide risk assessment support to project implementers through guidelines on financial risk management, and a Grant Risk Assessment and Management Tool.	Each WHO office maintains and develops Risk Registers (catalogues of risks that have been identified, assessed, and monitored).	An “In-Depth Review” risk assessment is conducted when a new program is established.
**Investigations and Audits**	Office of Audit and Investigations (OAI) conducts audits and investigations across the organization. Investigation results are submitted to the Administrator, senior management, and the External Board. The Secretary General is responsible for disciplinary proceedings. Audits are submitted to the Administrator, relevant Head of Bureau, the audited unit, and made publicly available.	The OIG is responsible for audits and investigations across the organization and its financed programs. Investigation and audit results are submitted to the Secretariat who determines the appropriate response.	Office of Internal Oversight Services (IOS) is responsible for internal audits and investigations. Investigation results are reported to the Director General, the Regional Director, and relevant responsible managers with recommendations for action. Audit reports are submitted to the External Auditor and Independent Expert Oversight Advisory Committee who are responsible for following up on recommendations. WHO is mandated to conduct an external assessment of the audit function by an independent reviewer at least once every 5 years.	The Integrity Vice Presidency’s Internal Investigations Unit (INT) is responsible for investigating allegations of sanctionable practices. Results of investigations are reported to the Evaluations and Suspension Officer who issues a Notice of Sanctions Proceedings, as well as relevant to national authorities. The Group Internal Audit (GIA) Vice Presidency conducts audits to determine whether processes for managing and controlling risks are designed and functioning effectively.

#### Risk assessments

UNDP defines risk as “uncertainty regarding the realization of organization goals” and states that “some of these risks may be ethical in nature, such as bribery, corruption, fraud, embezzlement, and kickbacks” [67]. UNDP addresses these risks through their *Enterprise Risk Management System* (ERM) (2019), whose scope and policies cover risks across all levels of the organization. Within their specific projects, *UNDP’s Policy on Fraud and Other Corrupt Practices* also identifies Project Managers as specifically responsible for identifying risks during the project design phase.

Within the Global Fund, the OIG is responsible for the risk assessments referred to them by the Secretariat [68]. These risk assessments are conducted by the Fund Portfolio Manager and relevant OIG representatives, who then make recommendations to the Director of Country Programs and the Inspector General [68]. The Global Fund also provides risk assessment support to project implementers, including guidelines on Financial Risk Management and a Grant Risk Assessment and Management Tool [69].

To manage risk, WHO offices are mandated to develop and maintain Risk Registers, which serves as a catalogue of risks that have been identified, assessed, and continue to be monitored [70]. Though the documents examined as part of this study held limited information on the WHO risk management process, the WHO identifies its *Good Governance for Medicines Program (which is no longer in place)* as a means of managing risk in the pharmaceutical sector at national levels, while its *Measuring Transparency in the Public Pharmaceutical Sector* provides a methodology to measure system vulnerabilities to risk.

The World Bank mandates a comprehensive risk assessment every time a new program is established. Their *In-Depth Review* risk assessment is described as a “multi-disciplinary tool that can be used to holistically and effectively address integrity risk in projects” [51]. Furthermore, the World Bank provides country-level support to conduct risk assessments for money laundering and terrorism as part of their commitment at the *UK Anti-Corruption Summit in 2016*. This support includes a tool that helps identify corruption risks in procurement and therefore aid agencies to proactively manage those risks [71].

#### Investigations and audits

Often, the responsibility of investigations and audits falls to a single department within an IO. Within UNDP, the *Office of Audit and Investigations* (OAI) is responsible for audits and investigations across the organization [72]. Results of investigations are submitted to the Administrator and other senior management as appropriate, as well as to the External board through an annual report [72]. Disciplinary processes based on the findings of investigations are the responsibility of the Secretary General [55]. The OAI is mandated to conduct proactive investigations when contacted by program managers about concerns related to the level of risk within a program [48]. The OAI also conducts internal audits with the goal of “improved effectiveness and efficiency of UNDP operations in achieving development goals” [73]. Internal audit reports are submitted to the Administrator, relevant Head of Bureau, and Head of the audited unit and are also made publicly available [73]. Though the OAI exercises “operational independence” in conducting its audits and investigations, the United Nations Board of Auditors is responsible for conducting external audits of UNDP programs and processes, which it submits to UNDP Executive Board [73]. The OAI performs audits in accordance with the International Standards for the Professional Practice of Internal Auditing [73].

Similarly, the Global Fund’s OIG holds responsibility for internal audits and investigations within the organization and its financed programs [74]. The OIG investigates allegations raised by the Secretariat or Ethics Office related to fraud, abuse, and corruption. The OIG then submits its reports to the Secretariat, who is responsible for determining and implementing the appropriate response [68]. The OIG also conducts audits of the Global Fund’s systems and processes, with its scope varying depending on the context [75]. Like UNDP, the OIG’s audits are in accordance with the Global Institute of Internal Auditors’ International Standards for the Professional Practice of Internal Auditing.

At the WHO, the *Office of Internal Oversight Services* (IOS) is responsible for internal audits and investigations. A report to the Internal Auditor stated that within the WHO, “investigations support WHO in managing risk of fraud and other wrongdoing by contributing to prevention, detection, and deterrence of wrongdoing including fraud, waste and abuse and providing risk-based, value-added, timely and result-oriented investigations” [76]. The IOS receives “Reports of Concern,” then proceeds with an investigation if reports are determined investigable [76]. Investigation results are then reported to the Director General, the Regional Director, and relevant responsible managers with recommendations for action. The IOS is also responsible for conducting internal audits, and does so in line with the Internal Auditors’ International Standards for the Professional Practice of Internal Auditing [76]. Audit reports are submitted to the External Auditor and the Independent Expert Oversight Advisory Committee, who utilize a web-based portal to manage and follow-up on audit recommendations [76]. The WHO is also mandated to conduct an external assessment of the audit function by an independent reviewer/review team at least once every five years [77]. Furthermore, the WHO has an External Auditor, appointed by and reporting to the World Health Assembly, who oversees the WHO’s operations, its financial risk management, and the efficacy of its internal control systems [78].

Finally, the World Bank has two separate institutions responsible for audits and investigations. The *Integrity Vice Presidency’s Internal Investigations Unit* (INT) is an independent unit responsible for investigating allegations of sanctionable practices [79]. The INT reports investigation results to the Evaluations and Suspension Officer, who then issues a Notice of Sanctions Proceedings to the relevant firm or individual [80]. Investigation reports are sent to regional operating staff in addition to the World Bank President. In order to prevent corruption in future operations, the World Bank also refers investigation findings to relevant national authorities when misconduct has violated a country’s laws [79]. The responsibility for conducting audits falls to the Group Internal Audit (GIA) Vice-Presidency who, like the other IOs, carries out audits in accordance with Internal Auditors’ International Standards for the Professional Practice of Internal Auditing [81]. Audits carried out by the GIA are meant to determine whether processes for managing and controlling risks are designed and functioning effectively [82].

### Enforcement and sanctions

Each of the IOs employ sanctions to enforce their anti-corruption agendas both within their organizations and vis-à-vis recipients of their funding and/or aid (see Table 6). All have sanctions processes through which those implicated in substantiated allegations of misconduct are punished.

**Table 6:** Enforcements and sanctions.

	UNDP	Global Fund	WHO	World Bank
**Enforcement and Sanctions Systems**	UNDP employees, non-employee affiliates, and UNDP vendors can be investigated if they are found to/suspected of engaging in fraud or corruption. If allegations are substantiated, the Secretary General or “officials with delegated authority” will impose sanctions.	The Global Fund has a sanctions process intended to protect the organization’s operations, resources, and reputation. As a result, if a Global Fund employee, supplier, or governance official engages in any “prohibited practice”, the Global Fund Secretariat can impose sanctions upon them. The Global Fund Sanctions Panel advises the Executive Direction of remedies for supplier misconduct.	WHO employees that fail to adhere to the organization’s code of conduct are investigated. If an alleged act of misconduct is substantiated, the employee may face any of the following disciplinary actions: a note of misconduct that is entered in the employee’s file, a fine, a grade reduction, suspension, or dismissal.	The World Bank has a sanctions system intended to protect the integrity of their operations and which serves to discourage individuals from engaging in sanctionable offences and promotes rehabilitation and prevention for those that do. Sanctions used within the Bank’s sanctions system include debarment, restitution, and/or reprimanding of the guilty party. Sanctions are imposed by the World Bank Sanctions Board.

Though sanctions processes exist across all IOs, the purpose of these procedures differs between organizations. While organizational documents indicate that the WHO has a sanctions procedure, there is a dearth of publicly available information about the specifics of this procedure. The World Bank states that the purpose of their sanctions procedure–created to contribute to the organization’s anti-corruption agenda–is deterrence rather than punishment, and that it ensures “that funds are used for their intended purposes” [79]. The World Bank Sanctions System serves to create both negative incentives to discourage the sanctioned party and others from engaging in future Sanctionable Practices, and positive incentives to encourage prevention, remediation and rehabilitation” [83]. At the Global Fund, however, the purpose of the sanctions process is to “…protect the interests, resources, and reputation of the…” organization [84]. Thus, the Fund’s sanctions process is focused more on mitigating reputational risk than deterring corruption. UNDP does not state an explicit purpose for its enforcement and sanctions processes.

At UNDP, authority to investigate allegations of misconduct from UNDP suppliers and impose sanctions against proven wrongdoers lies with the “Secretary-General or officials with delegated authority” who are internal to the organization [85]. Similarly, the sanctions process for UNDP vendors is conducted by *UNDP Vendor Review Committee*, another organization-affiliated body [48]. This suggests that sanctions, investigations, and processes are led by individuals who are, at least professionally, beholden to the organization. According to UNDP officials, this is the case since “a strong internal control system, where policies and procedures are enforced, internal controls are appropriately implemented, and staff members, non-staff personnel, vendors, implementing partners and responsible parties are informed about fraud and corruption and its consequences, can curtail fraud and corruption” [48].

The sanctions processes at the Global Fund and the World Bank are operated by a mix of internal and external individuals. The Global Fund’s sanction process is governed by the Global Fund Sanctions Panel, which “advises the Executive Director on remedies for supplier misconduct” and which is “made up of independent and senior management members of the Secretariat” [86]. Similarly, when allegations of sanctionable offenses by suppliers or vendors are made, the *World Bank Department of Institutional Integrity* (INT), an internal entity, investigates those allegations [80]. If the INT finds enough evidence to prove the occurrence of a sanctionable offence, it refers the case to the World Bank Evaluations and Suspension Officer (EO), another internal body [80]. The EO investigates the allegation and can impose sanctions upon the companies or individuals implicated. If the accused contest the allegations, the case is referred to the World Bank Sanctions Board, comprised of three internal and four external members [80]. The Sanctions Board then conducts an investigation into the allegations, before making a final judgement and, if appropriate, imposing sanctions [80].

While the Global Fund and the World Bank both have internal and external bodies involved in their sanctions processes, the World Bank’s three-tiered investigation system suggests that the World Bank prioritizes sanctioning as a mechanism for anti-corruption. This is further evidenced by the comparatively high number of documents published about the sanctions process at the World Bank as compared to the other studied organizations.

The World Bank, UNDP, and the Global Fund can debar suppliers found to have engaged in fraud or corruption. While debarment at each of these organizations serves a different purpose (in line with the intended outcome of the sanctions process as a whole), debarments are often made publicly available and can be imposed across multiple IOs. The World Bank has a cross-debarment agreement with UN agencies and with other international development banks, which “…ensures that sanctions have a powerful deterrent effect on the behavior of firms” [79]. Similarly, UNDP shares its list of debarred suppliers with other UN agencies and with the UN Global Marketplace, thus acting as a strong deterrent against engaging in acts of corruption [87]. The Global Fund, however, does not cross-debar, which could suggest that debarment from the Global Fund is less severe than debarment from the World Bank or UNDP. Furthermore, while most organizations keep records of currently and previously sanctioned suppliers, the Global Fund does not maintain a list of debarred suppliers. This means that the Global Fund is able to re-engage with previously-sanctioned vendors [86]. This calls into question the purpose of debarment from the Global Fund, as well as the efficacy of debarment as a method of achieving the purpose of the Fund’s sanction system, which is to “protect the interests, resources and reputation of the” organization [84].

### Reported outcomes

Though the terms outcome, output, and impact vary greatly in their meaning, the terms were used interchangeably and without definition across IOs. For clarity, we define output as the activities done by an organization, outcome as the observed effects of the outputs, and impact as the degree to which the observed outcome is attributable to the organization’s activities. The types of outcomes reported by the organizations are outlined below. The majority of these outcomes are quantitative and finance-based, such as amount of money lost to corruption, the number of investigations completed, and the number of parties sanctioned. The WHO and Global Fund additionally report on other outcomes, including increased transparency and accountability. The Global Fund, UNDP, and the World Bank do not elucidate the methods they use to measure the reported outcomes, unlike the WHO. An evaluation of how most anti-corruption mechanisms impact corruption is lacking across all organizations.

#### Outcomes of oversight mechanisms

The Global Fund, the WHO, and the World Bank monitor the impact of their oversight mechanisms through audits and evaluations and present the findings of these efforts in various reports. In their 2018 Annual Report, the Global Fund noted that the OIG’s audits and investigations helped the organization “identify and manage key risks, strengthen controls, improve processes, and ultimately deliver impact” [44]. The Global Fund’s *OIG Progress Report* also underscored that the OIG’s oversight assessments have allowed the OIG to attain information about and identify emerging fraud risks on an ongoing basis, leading to substantial financial recoveries for the organization [75].

In its *Report of the Internal Auditor*, the WHO’s internal controls were found to be operating effectively at their regional offices, headquarters, and global cross-cutting areas. Effectiveness was measured through a rating scale comprising the following options: “satisfactory,” “partially satisfactory,” or “unsatisfactory,” with the rating for the WHO improving from 70% to 100% between 2017 and 2018 [76]. However, the ratings for the country offices declined from 83% in 2017 to 60% in 2018, mainly due to 2018 audits capturing three “partially satisfactory, with major improvements needed” ratings (Country Offices in Chad, Ethiopia, and Somalia) and one “unsatisfactory” rating (Country Office in Yemen) [76]. This suggests that internal controls needed to be strengthened at country offices, especially those operating in resource-constrained environments.

In their 2019 report on their Sanctions System, the World Bank highlighted that this system has played a critical role in reviewing, investigating, and responding to allegations of fraud and corruption against firms or individuals participating in World Bank-financed projects [51]. In particular, findings from the *Joint In-Depth Fiduciary Review* helped identify potential indicators of procurement fraud and procedural non-compliance. It also contributed to the implementation of fraud and corruption mitigation measures [51]. In 2019, the Sanctions Board issued nine decisions pertaining to the cases it heard, including allegations of fraud, corruption, collusion, and obstruction [51].

#### Outcomes of anti-corruption initiatives

UNDP and the Global Fund report specifically on the country-level impact of their anti-corruption initiatives. For instance, UNDP’s *Results-Oriented Annual Report* (ROAR) finds that UNDP’s anti-corruption activities “are increasingly being mainstreamed and implemented as cross-cutting initiatives across UNDP’s thematic areas” [88]. In 2013, 82 UNDP country offices reported progress in implementing public sector anti-corruption frameworks and in strengthening transparent and accountable service delivery [88]. UNDP also reported positive outcomes of their social accountability initiatives. For example, in a Serbian initiative supported by GAIN, which aimed to promote anti-corruption within the health sector, integrated an SMS mechanism where citizens could file complaints pertaining to the corrupt practices they encountered. In 2014, 565 SMS reports were received, 310 of which were valid, 70% of which were resolved to the contentment of health service users [89].

UNDP reported several positive outcomes of their anti-corruption capacity building initiatives, with the main one being increased adoption of anti-corruption practices at the country-level [90]. As an example, UNDP’s “Workshop on Integrating Anti-Corruption into the UN Programming Process in Latin America and the Caribbean’’ held in Panama in 2013 showed that countries were making efforts toward anti-corruption. This included Uruguay’s plans to introduce anti-corruption principles in its new Country Programme Document, and Colombia’s incorporation of components highlighting transparency and accountability into their new United Nations Development Assistance Framework (UNDAFs) draft [90]. Additionally, over 50% of the country offices supported by UNDP’s GAIN have ongoing projects on anti-corruption/governance [90].

The Global Fund reported that their Pooled Procurement Mechanism, which aggregates orders and negotiates the best prices and delivery conditions for grant recipients, had increased access to information and mitigated health product procurement-related fraud [44]. The Global Fund also reported that their Wambo Platform had increased the transparency of the ordering process within the Pooled Procurement Mechanism [47]. The Global Fund reported significant progress toward transparency, as indicated by the following: (1) it achieved the highest possible transparency category in the 2016 Publish What You Fund Aid Transparency Index; (2) it was awarded the highest possible rating for transparency and accountability and overall organizational strength by the UK Department for International Development as part of their 2016 Multilateral Development Review; and (3) in 2017, the Multilateral Organization Performance Assessment Network (MOPAN) gave it top ratings in financial transparency and accountability [91].

## Discussion & conclusion

As demonstrated by the results above, there has been a significant increase in the adoption of anti-corruption mechanisms by IOs in recent years. This finding further corroborates other recent anti-corruption work including Lohaus & Gutterman [92], who determined that the overall quantity of anti-corruption commitments and support suggests that anti-corruption has become a robust international norm. Kohler & Bowra [26], who studied IOs generally, and Chang et al. [93], who focused on the Global Fund specifically, also demonstrated this marked increase over the past three decades. The increase in anti-corruption efforts can be attributed to a number of factors, including the increased public recognition of the negative financial, health, and development impacts of corruption. Public recognition has recently gained further traction as the COVID-19 pandemic has highlighted both the vulnerability of systems to corruption and the detrimental impacts that corruption can have, especially in times of crisis. Furthermore, the UNCAC, which was adopted in 2004, can be seen as an anti-corruption catalyst as it streamlines anti-corruption expectations at an international level. Finally, it is also important to note that the persistent advocacy work taken up by civil society organizations and scholars has pushed anti-corruption to the forefront of policy discussions and agendas around the world.

The findings of this study demonstrate that at this time, within the four IOs studied, corruption and risks of corruption are approached uniformly across sectors within each organization. More specifically, anti-corruption mechanisms are heavily focused on financial management, meaning that corruption is typically flagged through following the flow of funds throughout an initiative’s lifecycle. The financial focus of the anti-corruption efforts can be seen as a logical extension of the anti-corruption discourse born out of the body of economic literature that emerged in the 1990s [94]. This literature, which determined that corruption hinders development through wasting public resources [10, 23, 24, 95–100], was a driving influence of the international anti-corruption movement (as described above). However, as has been discussed by others including Bukovansky (2006) and Reinsberg et al. (2020; 2021), in focusing too closely on the economic drivers and implications of corruption, the underlying structural drivers of corruption remain unaddressed [101, 102]. Further, addressing corruption from an economic lens fails to address the specific risks, needs, and opportunities that exist in different sectors. As this study took a specific interest in the health sector, we found that though the IOs selected for this study were chosen based on their involvement in the health sector, there is a significant absence of anti-corruption mechanisms tailored to the specific and complex needs of the health sector.

While the increased number and scope of anti-corruption efforts in recent years has established a strong anti-corruption norm on the international stage, it is not yet possible to confirm whether IOs’ commitments to anti-corruption are authentic and effective. As described by Hafner-Burton & Schneider [103], anti-corruption rules and policies are not independently effective at addressing corruption without adequate monitoring and enforcement. It is difficult to discern the impact of the recent increased attention to corruption on the prevalence and severity of corruption within IOs and their programming. Though all studied IOs had monitoring and reporting mechanisms of some sort in place, as reported by Bauhr & Nasritousi [104], Coicaud [105], Chang et al. [93], and Kohler and Bowra [26], comprehensive and integrated evaluation strategies, techniques, and methodologies are missing from IO anti-corruption strategies. Moreover, the monitoring and evaluation strategies that are currently in place fail to measure the impact that anti-corruption mechanisms have on corruption itself, with corruption measured by proxy (e.g., through the level of transparency achieved or the number of times a reporting mechanism was utilized) rather than directly. Long-term, integrated evaluations are needed to determine if this increased attention to corruption through the implementation of ACTA mechanisms is effective at deterring and preventing actual instances of corruption.

In comparing anti-corruption mechanisms within and across IOs, it is important to note the associated difficulties. Cross-institutional comparison is difficult because there are no common anti-corruption definition and/or measurement standards. As can be seen from the results of this study, accountability, transparency, and corruption are defined and approached differently within each IO. This is in alignment with Rose-Ackerman’s findings that definitions of transparency and accountability are often overlapping and enmeshed in good governance agendas, which can obscure the intent of initiatives [106]. Further, IOs do not act in isolation, rather they are part of a global architecture that includes civil society organizations and governments. IOs and their anti-corruption efforts influence and are influenced by these other actors within this architecture which could account for some of the differences in anti-corruption approaches and mechanisms across organizations. With the recent emergence of the CATCH and other initiatives, however, there is the potential for improved collaboration between IOs to create shared definitions and evaluation standards.

IOs have played a key role in the fight against the COVID-19 pandemic and will continue to lead in these efforts as society aims to rebuild stronger health systems post-pandemic, making their commitment to anti-corruption essential. However, at the time of data collection, very little documents pertaining to the COVID-19 pandemic were identified within each of the four IOs. This may be due to the significant time it takes for policies to be changed or amended in large organizations such as the ones studied. However, given that public health emergencies increase risks of corruption, the need for comprehensive and effective anti-corruption strategies, in combination with monitoring and evaluation, is more important than ever.

While this paper examined anti-corruption mechanisms across IOs, there are two notable limitations to this analysis. Firstly, documents studied were limited to those published on organizational websites, meaning that external and potentially more critical sources and perspectives, such as media and academic literature, were not captured. Further, though beyond the scope of this paper, the critiques levied on the IOs’ rhetoric within their published documents are important to note. Such critiques include the detached, vague, and codified language often employed in IO documentation [107], and the pervasive gaps that exist between IOs’ talk, decisions, and actions [108]. However, authors mitigated this bias in part through the inclusion of reports and audits conducted by external bodies. Finally, this document analysis did not examine all audit and investigation materials produced by the IOs, nor did it capture materials that were not publicly available online. Rather, we explored recent reports and illustrative examples of the anti-corruption efforts made and mechanisms employed to begin to organize knowledge around the underexamined topic of anti-corruption efforts in global health systems.

This paper not only adds to the existing body of literature calling for increased monitoring and evaluation of anti-corruption efforts, but further advances knowledge of international anti-corruption processes in IOs. Through highlighting the strengths and gaps in various approaches, we have provided an in-depth initial overview of anti-corruption approaches employed by prominent IOs in the health sector. In doing so, we contribute knowledge on how best to address institutional corruption weaknesses for health systems strengthening. This research acts as a foundation for future research evaluating the efficacy of anti-corruption policies, institutions, and mechanisms, as well as the authenticity of anti-corruption commitments in IOs, both within and beyond the health sector.
